# Inhibition of Cochlear HMGB1 Expression Attenuates Oxidative Stress and Inflammation in an Experimental Murine Model of Noise-Induced Hearing Loss

**DOI:** 10.3390/cells10040810

**Published:** 2021-04-05

**Authors:** Cheng-Ping Shih, Chao-Yin Kuo, Yuan-Yung Lin, Yi-Chun Lin, Hang-Kang Chen, Hao Wang, Hsin-Chien Chen, Chih-Hung Wang

**Affiliations:** 1 Department of Otolaryngology-Head and Neck Surgery, National Defense Medical Center, Tri-Service General Hospital, Taipei 11490, Taiwan; eric660802@gmail.com (C.-P.S.); chefsketchup@hotmail.com (C.-Y.K.); yking1109@gmail.com (Y.-Y.L.); hwalongchen@yahoo.com.tw (H.-K.C.); meatball1027@yahoo.com.tw (H.W.); 2 Graduate Institute of Medical Sciences, National Defense Medical Center, Taipei 11490, Taiwan; lyc_1023@yahoo.com.tw; 3 Taichung Armed Forces General Hospital, Taichung 41168, Taiwan; 4 Graduate Institute of Microbiology and Immunology, National Defense Medical Center, Taipei 11490, Taiwan

**Keywords:** high-mobility group box 1 (HMGB1), cochlea, noise-induced hearing loss (NIHL), NADPH oxidase (NOX), reactive oxygen species (ROS), reactive nitrogen species (RNS), oxidative stress, inflammation

## Abstract

Noise-induced hearing loss (NIHL) is a common inner ear disease but has complex pathological mechanisms, one of which is increased oxidative stress in the cochlea. The high-mobility group box 1 (HMGB1) protein acts as an inflammatory mediator and shows different activities with redox modifications linked to the generation of reactive oxygen species (ROS). We aimed to investigate whether manipulation of cochlear HMGB1 during noise exposure could prevent noise-induced oxidative stress and hearing loss. Sixty CBA/CaJ mice were divided into two groups. An intraperitoneal injection of anti-HMGB1 antibodies was administered to the experimental group; the control group was injected with saline. Thirty minutes later, all mice were subjected to white noise exposure. Subsequent cochlear damage, including auditory threshold shifts, hair cell loss, expression of cochlear HMGB1, and free radical activity, was then evaluated. The levels of HMGB1 and 4-hydroxynonenal (4-HNE), as respective markers of reactive nitrogen species (RNS) and ROS formation, showed slight increases on post-exposure day 1 and achieved their highest levels on post-exposure day 4. After noise exposure, the antibody-treated mice showed markedly less ROS formation and lower expression of NADPH oxidase 4 (NOX4), nitrotyrosine, inducible nitric oxide synthase (iNOS), and intercellular adhesion molecule-1 (ICAM-1) than the saline-treated control mice. A significant amelioration was also observed in the threshold shifts of the auditory brainstem response and the loss of outer hair cells in the antibody-treated versus the saline-treated mice. Our results suggest that inhibition of HMGB1 by neutralization with anti-HMGB1 antibodies prior to noise exposure effectively attenuated oxidative stress and subsequent inflammation. This procedure could therefore have potential as a therapy for NIHL.

## 1. Introduction

Hearing handicaps arising from acoustic injury or noise trauma are a globally prevalent disability that manifests as hearing loss, tinnitus, the impairment of daily performance, and sleep disturbance [1]. More seriously, increasing numbers of young people are now suffering from recreational noise-induced hearing loss (NIHL) [2]. Complex pathological mechanisms give rise to the cochlear damage associated with NIHL. High-level impulse noise exposure often causes mechanical trauma, including disruption of the organ of Corti from the basilar membrane and rupture of the dendritic terminals of the auditory nerve fibers [3]. Steady-state noise exposure also causes metabolic overstimulation of factors like oxidative stress, inflammation, and apoptosis that are associated with NIHL [3,4,5].

Noise-associated oxidative stress in the cochlea is recognized as an important contributor to the pathogenesis of NIHL and may reflect a combination of overdriving of the mitochondria, glutamate excitotoxicity, and ischemia/reperfusion injury of the cochlear blood supply [3]. The end result of these processes is an increased generation of reactive oxygen species (ROS) and reactive nitrogen species (RNS) and subsequent cellular DNA and protein damage. Ultimately, these changes lead to damage to organelles and triggering of apoptotic/necrotic cell death [3,5].

A transient and intense ROS generation has been detected in the cochlea immediately after a noise exposure, suggesting a possible association between the initial hair cell damage and ROS formation. The cochlear ROS/RNS response may last for 2 weeks, with a maximum formation at 7 to 10 days after noise exposure, and this prolonged response contributes to long-term hair cell loss [6]. However, the molecular mechanism that leads to persistent ROS production is not yet clear.

One possible cell factor that may be involved in NIHL responses is the high-mobility group box 1 (HMGB1) protein. This is an abundant nuclear protein named for its high electrophoretic mobility on polyacrylamide gels. Immune activation, primary cell necrosis, or apoptosis can cause a release of HMGB1 from cells or its secretion by damaged cells and activated immune cells [7]. Extracellular HMGB1 is an important soluble factor that coordinates cellular events that are crucial for amplification of inflammation, for establishment of early immune responses, and even for tissue repair [8].

Extracellular HMGB1 functions as a proinflammatory cytokine and can trigger inflammatory responses upon binding to several cell-surface receptors, including the receptor for advanced glycation end products (RAGE) and the toll-like receptors TLR2, TLR4, and TLR9 [9]. Interestingly, several studies have shown that inhibition of HMGB1 expression with a neutralizing antibody can improve the severity of disease in models of sepsis, inflammatory diseases, and ischemia/reperfusion injuries [10,11,12,13,14]. HMGB1 also plays an important role in ROS generation [15,16,17,18], as RAGE transduces the signals of HMGB1 to enhance oxidative stress via NADPH oxidase (NOX) [16].

In a previous study, we reported that the increased expression of cochlear HMGB1 induced by NIHL was repressed by round window membrane-mediated dexamethasone treatment, suggesting that HMGB1 may be a useful marker of inflammation in NIHL [19]. However, the association between cochlear HMGB1 expression and oxidative stress in NIHL has not been fully explored.

In the present study, we hypothesized an involvement of the increased HMGB1 expression in the late production of ROS in noise-exposed cochleae. The purpose of this study was to investigate the effect of HMGB1 inhibition on cochlear ROS reduction and subsequent hair cell damage due to noise exposure.

## 2. Materials and Methods

### 2.1. Primary Cochlear Cell Culture

Cochleae of postnatal day 1 (P1) pups of CBA/CaJ mice were excised and transferred into Petri dishes containing phosphate buffered saline glucose solution (BSG; 116 mM NaCl, 27.2 mM Na2HPO4, 6.1 mM KH2PO4, 11.4 mM glucose). To prepare the dissociated cell cultures, 10 whole cochleae were pooled, cut into small pieces, and incubated in a mixture of 0.05% trypsin/0.02% (*w*/*v*) EDTA at 37 °C for 15 min, followed by repeated pipetting. The enzymatic digest was inactivated by adding 10% fetal bovine serum (FBS, Biological Industries, Beit Haemek, Israel) in a mixture of Dulbecco’s Modified Eagle Medium (DMEM, Gibco, Thermo Fisher Scientific Inc., Waltham, MA, USA). Tissue dissociates were filtered through a 40 mm mesh to remove cell aggregates and debris. These newly dissociated primary cochlear cells were plated in 6 cm dishes at 37 °C in a 5% CO_2_ atmosphere in DMEM supplemented with 10% FBS and penicillin-G. The primary cells were subcultured every 3 days. The secondary or tertiary cells were used for the following experiments.

### 2.2. Immunofluorescence Staining of 4-HNE

The primary cochlear cells were treated with recombinant HMGB1 protein (BioVision, San Francisco, CA, USA) for 24 h and the immunofluorescence staining of 4-HNE was performed for ROS detection. Cells incubated with lipopolysaccharide (LPS, 0.1 μg/mL) were used as positive control. The cells were washed twice with phosphate buffered saline (PBS) and fixed with freshly prepared 4% paraformaldehyde at 37 °C for 30 min. The cells were permeabilized with 0.1% Triton X-100 for 5 min in BlockPRO blocking buffer (Visual Protein Biotechnology, Taipei, Taiwan). After PBS with Tween 20 (PBST) washes, nonspecific antibody binding was blocked by BlockPRO blocking buffer for 60 min at room temperature (RT). The cells were incubated with polyclonal primary antibodies to 4-HNE (1:100; Abcam, Cambridge, UK) in an antibody dilution buffer (Dako, Agilent Technologies, Inc., Santa Clara, CA, USA), incubated in a humidified chamber for 1 h at RT, and, after being washed with PBST, stained with a secondary antibody (Donkey anti-rabbit Alexa Fluor 488, 1:500, Molecular Probes, Thermo Fisher Scientific Inc., Waltham, MA, USA) for an additional 60 min. After being washed three times with PBST, the cells were mounted in 4,6-diamidino-2-phenylindole (DAPI) Fluoromount-G^®^ mounting medium (SouthernBiotech, Birmingham, AL, USA). Cell images were captured with an LSM 880 Zeiss confocal microscope.

### 2.3. Quantitative PCR for Analysis of iNOS Gene (Nos2) Expression

Primary cochlear cells were seeded on 6-well plates overnight, then were treated with recombinant HMGB1 for 72 h. Total RNA of each samples using high pure RNA isolation kit (F. Hoffmann-La Roche Ltd., Basel, Switzerland) were extracted from primary cochlear cells. RNA was converted to cDNA using QuantiNova Reverse Transcription kit (QIAGEN GmbH, Hilden, Germany). Gene expression was measured with TaqMan gene expression assays (Thermo Fisher Scientific Inc., Waltham, MA, USA) for Nos2 (iNOS gene; probe ID: Mm00440502_m1) and Gapdh (probe ID: Mm99999915_g1) using QuantiNova Probe RT-PCR Kit (QIAGEN GmbH, Hilden, Germany) and a QuantStudio 5 Real-Time PCR system (Thermo Fisher Scientific Inc., Waltham, MA, USA). The qPCR data were presented as gene expression level relative to the level of no treatment controls after normalization with the expression of GAPDH.

### 2.4. Animals and Study Design

The experimental protocol was approved by the Institutional Animal Care and Use Committee of the National Defense Medical Center, Taipei, Taiwan. Animal care complied with all institutional guidelines and regulations. A total of 60 CBA/CaJ mice aged from 4 to 8 weeks and 5 P1 pups of CBA/CaJ mice were used. In the first in vivo experiment, 28 mice were exposed to noise and then were sacrificed at post-noise days 1, 4, 7, and 14 for immunohistochemistry and Western blot analysis. In the second experiment, 32 mice were separated into two study groups. The experimental group received an intraperitoneal injection of polyclonal chicken IgY anti-HMGB1 antibody (2 mg/kg in 0.1 mL saline [20]; Sagamihara, Kanagawa, Japan) 30 min before noise exposure. The control group was injected with 0.1 mL saline only.

### 2.5. Noise Exposure

Mice were anesthetized, placed in a soundproof booth with a loudspeaker (V12 HP; Tannoy, Ltd., Coatbrige, UK) mounted above the center of the cage, and exposed to 110 dB sound pressure level (SPL) white noise for 3 h. A specially designed and separated wire cage was used to avoid inappropriate exposure to noise caused by the animals congregating during the noise stimulation. The noise level was measured using a sound level meter (Rion NL-52, Tokyo, Japan), and differences in the noise level within the booth near the center or edge of the cage were less than 1 dB.

### 2.6. Tissue Preparation and Immunohistochemistry

The cochleae were dissected and perfused with 4% paraformaldehyde (in 0.1 M phosphate buffered saline [PBS], pH 7.4) through the opened oval window and a small hole in the apex. After a 2 h post-fixation, the cochleae were decalcified in 10% ethylenediaminetetraacetic acid (EDTA), pH 7.3, at 4 °C on a rotating shaker; the EDTA solution was changed daily until decalcification was complete. After immersion in a graded sucrose series (15, 20, and 25%) for 30 min, and overnight immersion in 30% sucrose at 4 °C, the cochleae were embedded in paraffin, sectioned at 5 μm, and immunostained using the Mouse/Rabbit PolyDetector HRP/DAB Detection System (Bio SB Inc., Santa Barbara, CA, USA) and standard procedures for quenching (H_2_O_2_ in methanol) and blocking (PolyDetector Peroxidase Blocker) endogenous peroxidase activity and for blocking non-specific antibody binding (1× PBS containing 3% horse serum and 0.3% Triton™ X-100). The slides were blotted, covered with antibody dilution buffer (Dako Co., Carpinteria, CA, USA) containing mouse anti-HMGB1 (1:50; Novus Biologicals, Littleton, CO, USA), anti-4-hydroxynonenal (anti-4-HNE) (1:50; Abcam, Cambridge, UK), anti-nitrotyrosine (1:50; Santa Cruz Biotechnology, Santa Cruz, CA, USA), anti-inducible nitric oxide synthase (iNOS, 1:200; Novus Biologicals, Centennial, CO, USA), or anti-intercellular adhesion molecule-1 (ICAM-1; 1:200; eBioscience, Inc., San Diego, CA, USA), and incubated in a humidified chamber for 2 h at RT, washed, and incubated with PolyDetector Label HRP secondary antibody for 30 min at RT. The slides were washed again, treated with PolyDetector DAB substrate-chromogen solution for 10 min, rinsed in distilled water, counterstained with hematoxylin (Muto Pure Chemicals Co., Ltd., Tokyo, Japan), dehydrated through a graded alcohol series (50–100%), cleared in xylene, and mounted in Permount (Fisher Scientific, Pittsburgh, PA, USA). The slides were examined using an Olympus BX50 microscope equipped with a digital camera (Olympus DP74, Olympus Corp., Tokyo, Japan).

### 2.7. Cochlear Cryosections and Immunostaining

Following gentle perfusion of 4% paraformaldehyde into the cochleae, the cochleae were post-fixed for 2 h at RT, decalcified and dehydrated as described above, and then transferred to OCT embedding medium. A stereomicroscope was used to orient the cochleae, and the samples were transferred into a freezing slurry of solid CO_2_ and 100% ethanol for 7–10 s until tissue was immobilized and solidified. Using a cryostat microtome, 10 μm mid-modiolar sections of frozen cochleae were cut and mounted on glass slides. For immunostaining, the slides were incubated with anti-NOX4 polyclonal antibodies (1:100; Abcam, Cambridge, UK) for 2 h. After three washes with PBS, the slides were incubated with Alexa-Fluor-555-conjugated goat anti-rabbit antibodies (1:500; Thermo Fisher Scientific, Eugene, OR, USA) for 1 h. After rinsing with PBS, the samples were mounted in 4,6-diamidino-2-phenylindole (DAPI) Fluoromount-G^®^ mounting medium (SouthernBiotech, Birmingham, AL, USA), and covered with a coverslip. Fluorescence images were obtained using an Olympus BX50 microscope. Immunostaining was quantified by analyzing all camera images using the open-source ImageJ, Version 1.52v Fiji software (https://imagej.net/Fiji.html#Downloads, accessed on 4 April 2021). The staining intensities were expressed in arbitrary units (AU) for the different cochlear cell types and subjected to histogram analysis.

### 2.8. Cochlear Surface Preparations and Outer Hair Cell Survival Rate

Mice were flushed with pre-warmed PBS and then transcardially perfused with 4% paraformaldehyde. The cochleae were removed, and the oval window and the cochlear apex were opened to facilitate immediate perfusion with 4% paraformaldehyde in PBS. The samples were then immersed in the same fixative solution for 1 h at RT to allow diffusion through the whole cochlea. After washing with PBS, the bony capsule surrounding the cochlea, the cochlear lateral wall, and Reissner’s membrane were removed. The remaining part of the cochlea was permeabilized with 0.3% Triton X-100 for 10 min, followed by staining with 2% Alexa Fluor 488-conjugated phalloidin (Molecular Probes) for 40 min. After three washes with PBS, the entire basilar membrane containing the organ of Corti was dissected into half turns, whole mounted on glass slides with DAPI Fluoromount-G^®^ mounting medium, and covered with a coverslip for analysis. The flat surface preparation of the organ of Corti was examined over its entire length with an Olympus BX50 microscope. The survival rates of the outer hair cells (OHCs) were calculated as described previously [19].

### 2.9. Western Blotting

Aliquots of cochlea homogenates were separated on 8% sodium dodecyl sulfate (SDS) polyacrylamide gels, transferred to polyvinylidene difluoride (PVDF) membranes (Millipore, Billerica, MA, USA), blocked with 5% skimmed milk in TBST (0.2 M Tris-base, 1.37 M NaCl, and 0.1% Tween 20), probed with the indicated primary antibody at 4 °C overnight, washed with TBST, and incubated with anti-rabbit horseradish peroxidase-linked whole antibody (1:10,000; GE Healthcare, Chicago, IL, USA) for 1 h at RT. The immunoreactive bands were stained using a light emitting nonradioactive method (ECL; Millipore). The specific primary antibodies were anti-HMGB1 antibody (1:1000; Novus Biologicals, Littleton, CO, USA), anti-4-HNE antibody (1:1000; Abcam, Cambridge, UK), and anti-actin antibody (1:1000; Millipore, Burlington, MA, USA).

### 2.10. Auditory Brainstem Response Recording

Auditory function was evaluated by recording auditory brainstem responses (ABRs) in anesthetized mice, as described previously [19]. Specific stimuli (clicks and 8-, 16-, 32-kHz tone bursts) were generated by using SigGen software (Tucker-Davis Technologies, Gainesville, FL, USA) and delivered to the external auditory canal. The average responses from 1024 stimuli for each frequency were obtained by reducing the sound intensity in 5 dB steps until threshold. The resulting ABR thresholds were defined as the lowest intensity at which a reproducible deflection in the evoked response trace could be recognized.

### 2.11. Statistical Analysis

Statistical analysis was performed using a two-tailed Student’s *t*-test. Results are expressed as the mean ± standard error of the mean (SEM). Differences were considered significant at *p* < 0.05.

## 3. Results

### 3.1. Recombinant HMGB1 Activated 4-HNE Production and Induced the Expression of iNOS Gene in Primary Cochlear Cells

**Figure 1:**
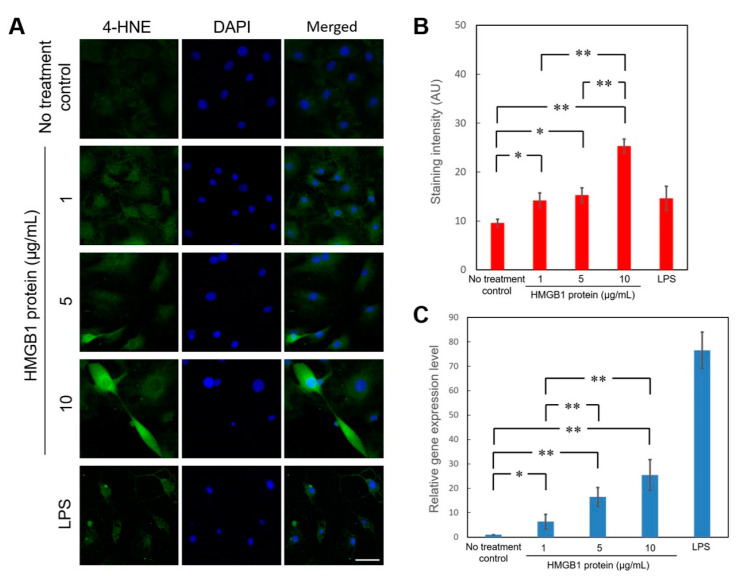
Recombinant HMGB1 activated 4-HNE production and induced the expression of iNOS gene in primary cochlear cells. (**A**) After incubation with various concentrations of recombinant HMGB1 for 24 h, immunostaining for 4-HNE was used to determine the generation of reactive oxygen species in primary cochlear cells. Representative immunofluorescence staining for 4-HNE (green), DAPI (blue), and merged images in the cells treated with recombinant HMGB1 or LPS. (**B**) Histogram representations of mean fluorescence intensity of 4-HNE staining intensities. Data are shown as the means ± SEM (*n* = 6 for each bar). Scale bars = 50 μm. (**C**) Recombinant HMGB1 stimulated the expression of iNOS gene (NOS2) in primary cochlear cells. Gene expression level was determined by quantitative PCR and expressed as the level relative to no treatment controls. Data are shown as the means ± SEM (*n* = 5 for each bar). * *p* < 0.05; ** *p* < 0.01; 4-HNE = 4-Hydroxynonenal; DAPI = 4,6-diamidino-2-phenylindole; LPS = lipopolysaccharide; SEM = standard error of the mean.

We first examined whether cochlear cells respond to excessive HMGB1 to initiate subsequent ROS activation. As shown in Figure 1A,B, a different concentration of recombinant HMGB1 treatments resulted in a dose-dependent induction of 4-HNE in cochlear primary cultured cells. Besides, recombinant HMGB1 also upregulated the iNOS (NOS2) gene expression in primary cochlear cells with a dose-dependent effect (Figure 1C). These results implicated that inner ear sensory organs might be targeted by HMGB1-mediated inflammation or oxidative stress that contributes to cochlear injury after noise exposure.

### 3.2. Noise Exposure Increased Cochlear HMGB1 Expression and Oxidative Stress

**Figure 2:**
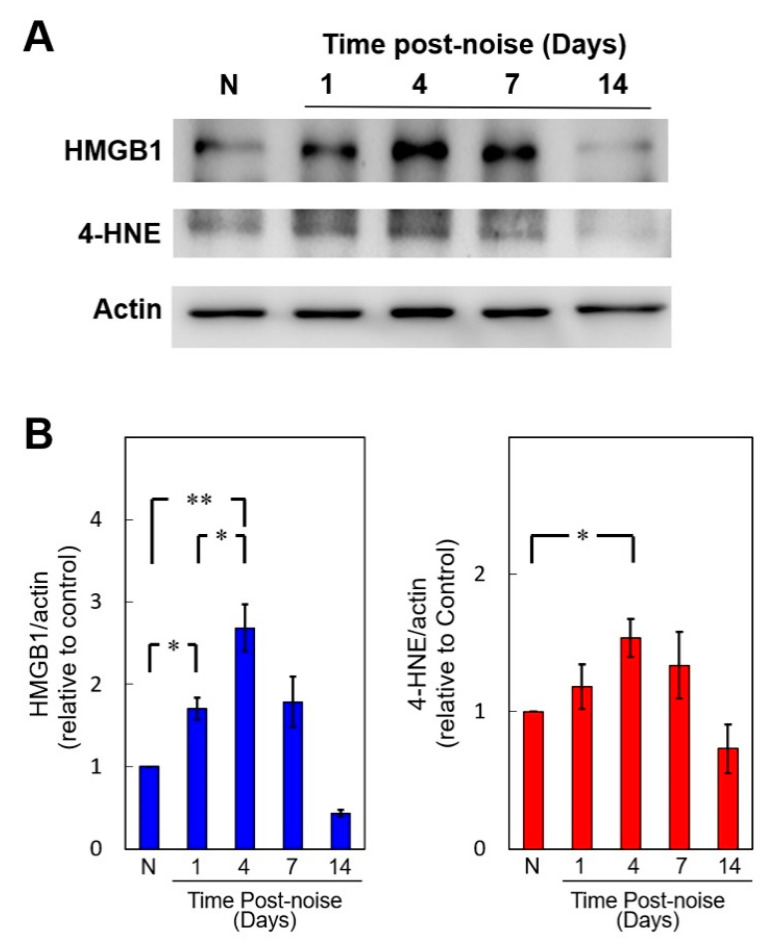
Noise exposure upregulates cochlear expression of high mobility group box 1 (HMGB1) and 4-HNE. (**A**) Western blot analysis of cochlear HMGB1 and 4-HNE expression after noise exposure. (**B**) Quantification of the time course of cochlear HMGB1 and 4-HNE expression (*n* = 4 [refers to 8 cochleae from 4 animals] for each bar). The results are expressed as the mean ± SEM. * *p* < 0.05; ** *p* < 0.01; N = a control mouse group not exposed to noise; SEM = standard error of the mean.

Figure 2 shows that both HMGB1 and 4-HNE were upregulated in the mouse cochleae at different time points after noise exposure. The HMGB1 and 4-HNE levels progressively increased beginning at post-exposure day 1 and reached a maximum at day 4 (Day 4 vs. control, *p* = 0.0098 in HMGB1 and *p* = 0.039 in 4-HNE) (Figure 2B). On post-exposure day 7, significant overproduction of both HMGB1 and 4-HNE was still evident in the noise-exposure group but not in the control group (Day 7 vs. control, *p* = 0.044 in HMGB1 and *p* = 0.013 in 4-HNE), although both levels had dropped from the day 4 levels. The increased levels in the noise-exposure group recovered to baseline levels on day 14. The similarity of the time-dependent changes in HMGB1 and ROS generation suggested a positive correlation between the HMGB1 levels and oxidative stress in response to a cochlear noise insult.

**Figure 3:**
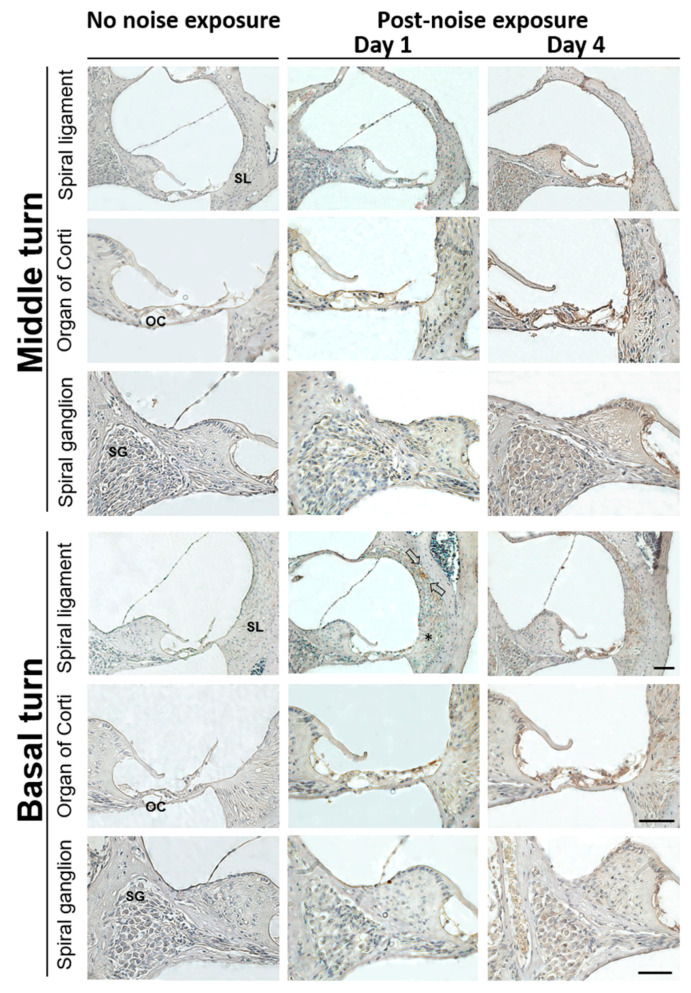
Representative expression and distribution of HMGB1 in mouse cochlear tissues following noise exposure. HMGB1 immunohistochemical staining (brown color) was substantially increased on post-exposure day 1 in the spiral ligament of the basal turn. Arrows indicate HMGB1-positive staining cells, mainly localized in the type II cell region. On day 4, the HMGB1 was markedly expressed in the organ of Corti, spiral limbus, spiral ganglion, and spiral ligament of the cochlear basal and middle turns (*n* = 4 [refers to 4 cochleae from 4 different animals]). Scale bars = 50 μm. SL = spiral ligament; OC = organ of Corti; SG = spiral ganglion.

Immunohistochemistry was also used to analyze the distribution of HMGB1 expression in the cochlea after noise exposure (Figure 3). On post-noise exposure day 1, a local increase was noted for HMGB1 immunostaining in the spiral ligament of the cochlear basal turn, mostly at the region characterized by type I and II fibrocytes (Figure 3, arrows). On day 4, a markedly increased HMGB1 expression was evident in the cochlear middle and basal turns, including the organ of Corti, spiral ligament, spiral limbus, and spiral ganglion regions, with the most intense HMGB1 expression occurring in the organ of Corti and spiral ligaments. Therefore, noise exposure appeared to induce cochlear HMGB1 expression starting at post-exposure day 1, with possible initiation in the spiral ligament.

### 3.3. Pretreatment with Anti-HMGB1 Neutralizing Antibody Attenuated HMGB1 Expression, ROS Generation, and Subsequent Inflammation in Noise-Exposed Cochlea

**Figure 4:**
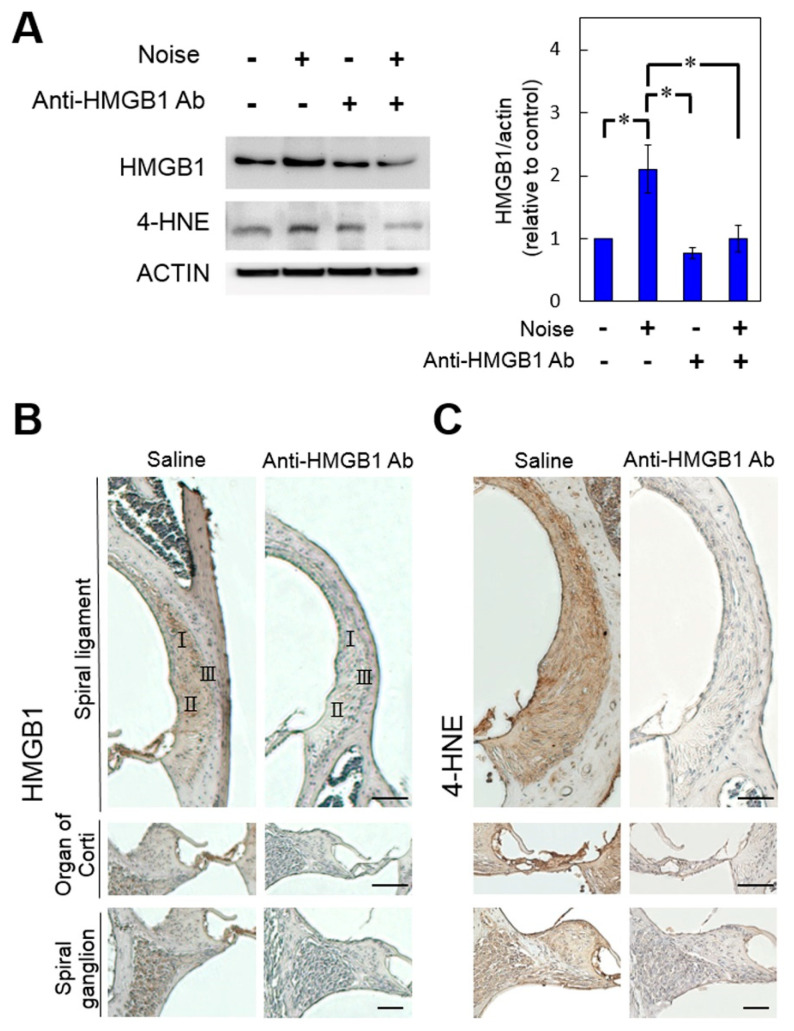
Blockade of HMGB1 by pre-treatment with anti-HMGB1 antibodies diminished the noise-induced increases in ROS in the cochlea. Mice were intraperitoneally treated with anti-HMGB1 antibodies or saline 30 min prior to noise exposure. Samples of cochlear homogenates or sections were collected 4 days after noise exposure. (**A**) Western blot analysis for HMGB1 and 4-HNE in the cochleae treated with anti-HMGB1 antibodies or saline (*n* = 4 [refers to 8 cochleae from 4 different animals] for each bar). Representative immunohistochemical staining (brown color) for (**B**) HMGB1 and (**C**) 4-HNE of the cochleae (*n* = 4 [refers to 4 cochleae from 4 different animals]). Scale bars = 50 μm. * *p* < 0.05.

As shown in Figure 4A, in the absence of noise exposure, administration of anti-HMGB1 antibodies appeared to partially neutralize the cochlear HMGB1 but had no significant effect on 4-HNE expression or on the basal level of ROS in the cochlea. By contrast, pretreatment with anti-HMGB1 antibodies following noise exposure not only attenuated the elevated cochlear HMGB1, but it also decreased the elevation of 4-HNE following noise exposure. The immunohistochemical analysis results (Figure 4B,C) agreed with the Western blot findings.

**Figure 5:**
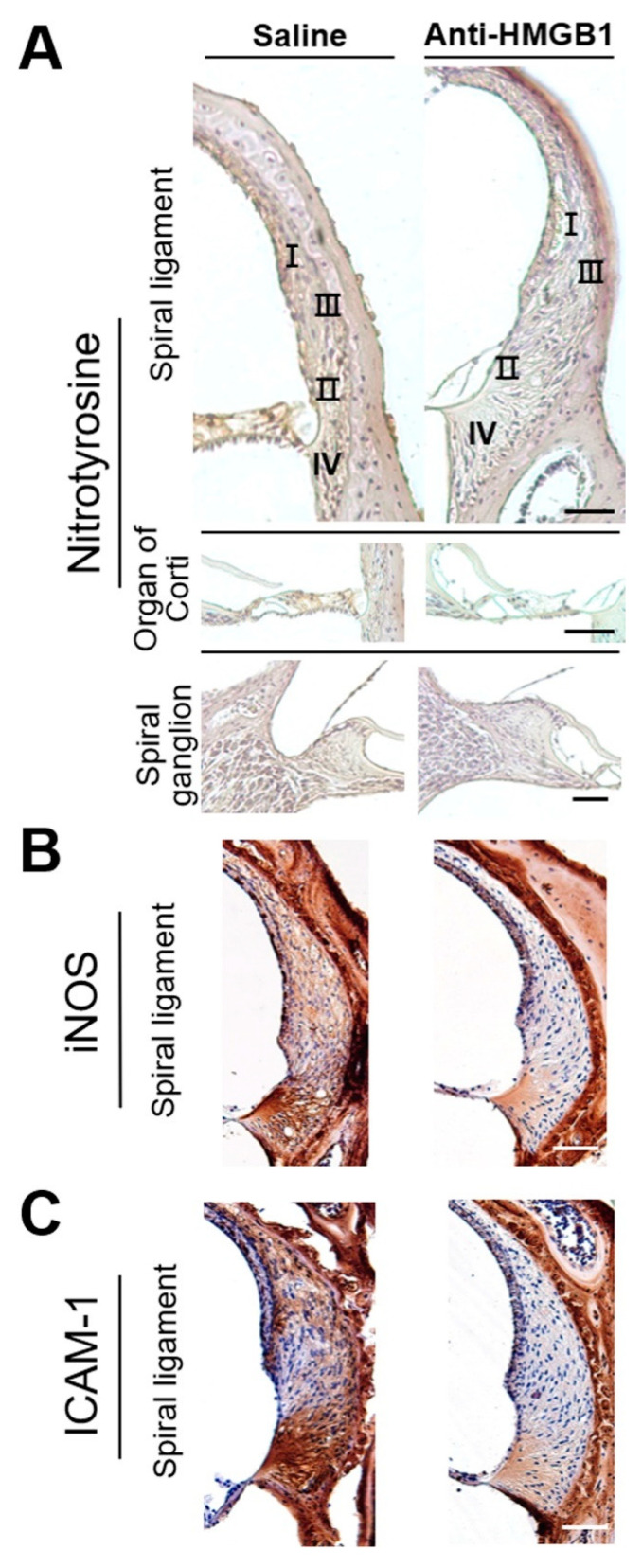
Blockade of HMGB1 by pretreatment with anti-HMGB1 antibodies diminished the noise-induced increase in RNS level and inflammation in the cochlea. Mice were treated intraperitoneally with anti-HMGB1 antibodies or saline at 30 min prior to noise exposure. Samples of cochlear homogenates or sections were collected 4 days after noise exposure. Representative immunohistochemical staining (peroxidase/DAB (brown color) for (**A**) nitrotyrosine, (**B**) iNOS, and (**C**) ICAM-1 in the cochlea (*n* = 4 [refers to 4 cochleae from 4 different animals]). Sections were counterstained with hematoxylin. I, II, III, IV = classification of spiral-ligament fibrocytes. Scale bars = 50 μm.

Increased nitric oxide (NO) levels have been recognized to contribute to oxidative stress in the cochlea following acoustic trauma [21,22,23]. Figure 5 shows that both nitrotyrosine (Figure 5A), a biomarker of RNS, and iNOS (Figure 5B) immunostaining were significantly diminished from the control levels in the mouse cochlea by pretreatment with anti-HMGB1 group on post-exposure day 4. Therefore, inhibition of noise-induced HMGB1 appeared to repress noise-induced activity of iNOS and RNS generation. 

ICAM-1, which functions as a proinflammatory cytokine in the recruitment of leukocytes to the cochlea, was upregulated following acoustic exposure [19,24]. HMGB1 plays a significant role in the response to noise-induced cochlear inflammation [19]; however, the effect of anti-HMGB1 on cochlear ICAM-1 after noise exposure has not been tested previously. Noise exposure increased the intensity of ICAM-1 expression, but this increase was suppressed by pretreatment with anti-HMGB1 antibodies (Figure 5C).

**Figure 6:**
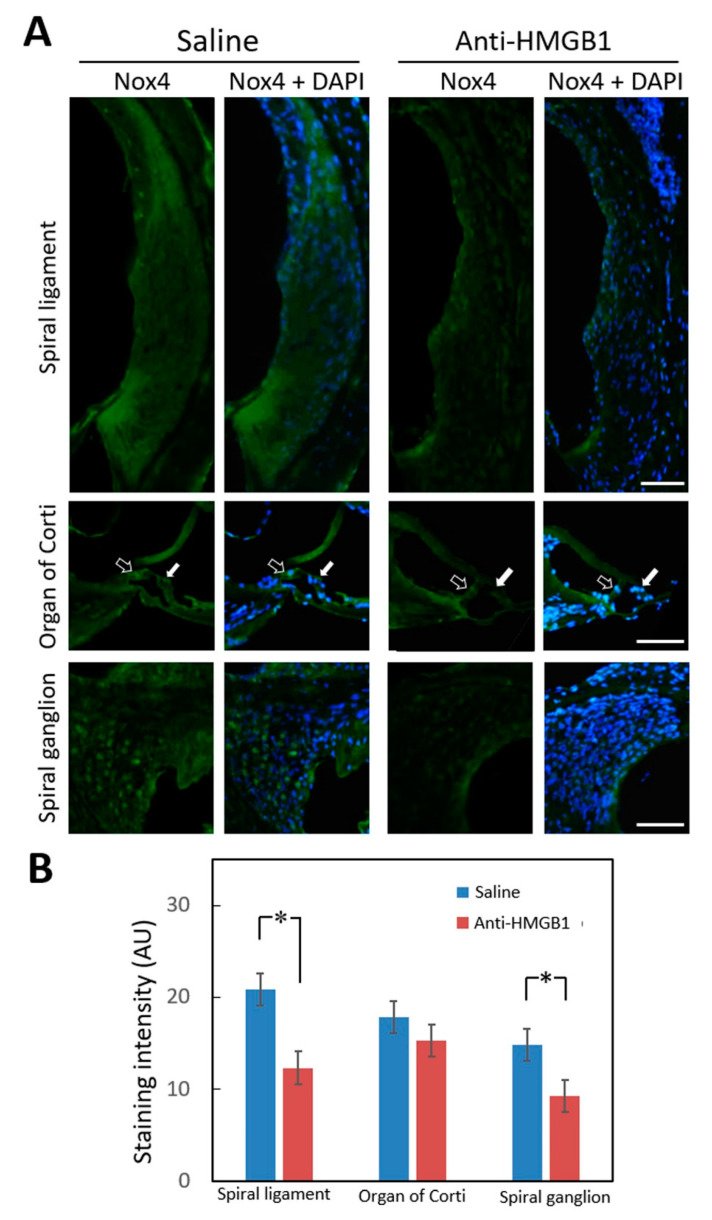
Immunohistochemical staining for NOX4 in the cochlea 4 days after noise exposure. (**A**) Representative staining of cochlear sections from mice pretreated with anti-HMGB1 antibodies or saline. Hollow arrows indicate inner hair cells and white arrows indicate outer hair cells. Labeling: Nox4 (green); DAPI (blue). (**B**) Histogram representations of the mean fluorescence intensity of Nox4 staining. Data are shown as the means ± SEM (*n* = 4 [refers to 4 cochleae from 4 different animals]) for each bar). Scale bars = 50 μm. * *p* < 0.05; Nox4 = NADPH oxidase 4; DAPI = 4,6-diamidino-2-phenylindole; SEM = standard error of the mean.

Treatment with neutralizing anti-HMGB1 antibodies prior to noise exposure therefore appeared to inhibit noise-induced ROS generation in the cochlea. NADPH oxidase 4 (NOX4), which is one member of the NOX family that generates ROS, has been detected in the mouse cochlea. Overproduction of NOX4 in a transgenic mouse model rendered the mice vulnerable to NIHL [25]. HMGB1 mainly binds to RAGE and TLR4 [26], and NOX4 can also directly interact with RAGE and TLR4 [17,27,28,29]. Figure 6 shows that anti-HMGB1 pretreatment significantly reduced noise-induced NOX4 expression, especially in the spiral ligament (*p* = 0.042) and spiral ganglion (*p* = 0.037) regions. In the region of the organ of Corti, no significant difference was found between the experimental and control groups (*p* = 0.383); however, the expression of NOX4 in the inner and outer hair cells was lower in the anti-HMGB1 group than in the control group (Figure 6A). Therefore, neutralizing the elevated HMGB1 levels caused by noise exposure also repressed NOX4 expression in the cochlea.

### 3.4. Suppression of HMGB1 Expression Ameliorated NIHL and Cochlear Hair Cell Loss

**Figure 7:**
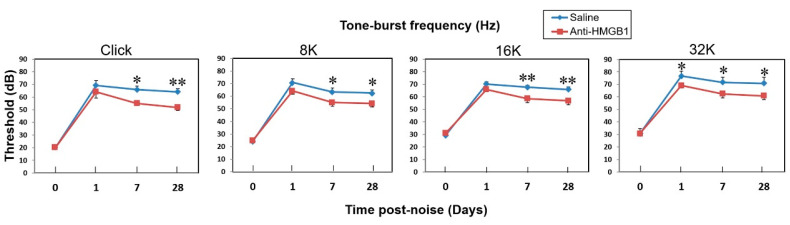
Neutralizing anti-HMGB1 antibodies reduced the auditory brainstem response (ABR) threshold shift in mice with noise-induced hearing loss. The ABR was recorded from one ear in each animal. The results are expressed as the mean ± SEM (*n* = 5 [refers to 5 measured ears from 5 different animals]). * *p* < 0.05; ** *p* < 0.01; SEM = standard error of the mean.

We evaluated the hearing function of mice prior to and at 1, 7, and 28 days after noise exposure by measuring the ABR to click and tone-burst stimuli (Figure 7). At post-noise day 1, no significant differences were detected in the ABR between the anti-HMGB1-treated and saline-treated groups, except at the 32 kHz frequency. At 7 days post noise, significantly fewer threshold shifts were seen in the anti-HMGB1 treatment group than in the saline group at click and frequencies crossing the low to high tone-burst stimuli. Therefore, blocking the signal pathway of HMGB1 via anti-HMGB1 antibodies appeared to rescue hearing loss in mice that had suffered a noise trauma.

**Figure 8:**
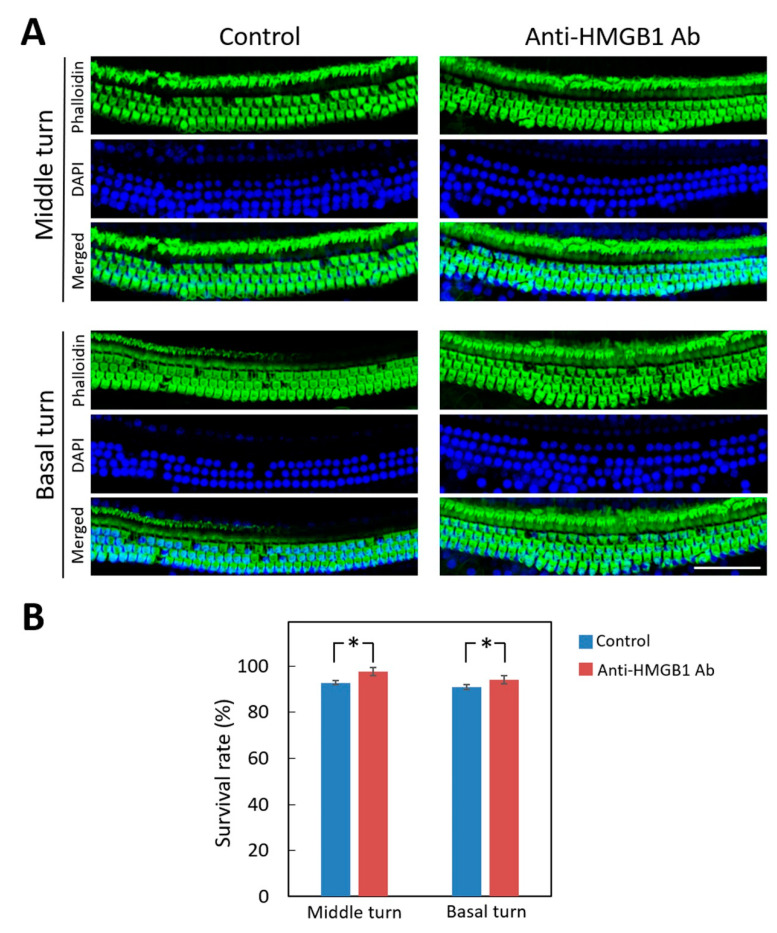
Pretreatment with anti-HMGB1 antibodies protects auditory hair cells in noise-exposed cochlea. (**A**) Representative images of a surface preparation of the basal and middle turns of the cochlea of the pretreated and untreated control groups on day 28 after noise exposure. Immunofluorescence staining shows the nuclei (blue, DAPI) and filamentous actin (green, phalloidin). (**B**) The survival rates of the outer hair cells in the basal and middle turns of mouse cochleae from each group. The results are expressed as the mean ± SEM (*n* = 4 [refers to 4 cochleae from 4 different animals] for each bar). Scale bars = 50 μm. * *p* < 0.05; DAPI = 4,6-diamidino-2-phenylindole; SEM = standard error of the mean.

Figure 8 shows the severity of hair cell loss in the cochlea from the different treatment groups at day 28 after noise exposure. The preservation of hair cells was significantly greater in the mice pre-treated with anti-HMGB1 antibodies than with saline and especially in the middle and basal turns of the organ of Corti. No significant hair cell loss was observed in the apical turn in either group. Therefore, inhibition of elevated HMGB1 expression could protect the cochlea from noise-induced damage.

## 4. Discussion

The current study examined the time-course changes in HMGB1 and ROS production in the noise-exposed cochlea for a period of up to 2 weeks. Dynamic changes were observed in cochlear HMGB1 expression, beginning with an immediate upregulation at post-noise day 1, a maximum at day 4, a decline at day 7, and then a recovery to the pre-noise level at day 14. Interestingly, Ladrech et al. found that aminoglycoside-injured organs of Corti in the rat also had the highest perilymphatic HMGB1 concentrations at 4 days after the treatment [30]. To date, only two published studies have directly addressed and measured the HMGB1 in an experimental NIHL model [19,31]. The first study, by Chen et al., reported changes in several inflammatory mediators in a guinea pig NIHL model involving treatment with hydrogen-saturated saline [31]. Although they concluded that HMGB1 does not seem to be involved in the pathogenesis of NIHL, their observations might be limited by the use of cross-sectional measurement in their study design. The second study was our previous work on measurements of HMGB1 in guinea pigs pretreated with dexamethasone. We found a marked suppression of the cochlear inflammatory response and a decrease in the expression of ICAM-1 and HMGB1 in noise-damaged cochlea [19].

ROS can be generated immediately after noise exposure and can undergo continuous induction in the cochlea for 7–10 days [5,6]. Consequently, ROS can serve as a marker for investigating interventions that involve the neutralization of HMGB1. Our results supported the hypothesis that cochlear HMGB1 may directly or indirectly contribute to ROS formation in noise-exposed cochlea. This study is the first to indicate a correlation between cochlear oxidative stress and HMGB1 expression in NIHL.

Noise trauma is also known to promote the expression of nitric oxide synthase enzymes (notably iNOS) and the generation of nitric oxide (NO) in the cochlea [5,21]. The NO produced through the enzymatic reaction of inducible nitric oxide synthase 2 (NOS2) can react with superoxide (O_2_^−•^), a ROS formed enzymatically by NADPH oxidase, to generate cytotoxic reactive nitrogen species (RNS). Both ROS and RNS are thought to play a major role in tissue oxidative damage and dysfunction. Treatment with an inhibitor of iNOS to reduce NO generation has been shown to improve NIHL [22,23]. The genes for iNOS can also be regulated by HMGB1 [32], although most of the evidence regarding the role of HMGB1 in cochlear iNOS expression comes from cisplatin ototoxicity [15,33].

Cisplatin not only increases the transcriptional and translational expression of TLR4 in the cochlea, but it also increases the interaction between TLR4 and LPS, thereby upregulating the production of several proinflammatory cytokines, such as TNF-a, IL-1b, and IL-6, via nuclear factor (NF)-kB activation [33]. The interaction between HMGB1 and a TLR2 agonist (e.g., peptidoglycan) or a TLR4 agonist (e.g., LPS) has a synergistic effect on iNOS expression and NO release by upregulating greater numbers of surface receptors (TLR2/4 and RAGE). This increase in receptors, in turn, amplifies the activation of MAPKs (p38 and JNK) and NF-κB, thereby enhancing iNOS expression and NO production [34].

For noise-exposed cochlea, the role of HMGB1 is unclear and needs further elucidation. HMGB1, as a highly conserved and ubiquitous protein in the nucleus and cytoplasm of nearly all cell types, may also possibly participate in noise-induced cochlear insults. In the present study, nitrotyrosine, a marker of cell inflammation as well as of NO production, was markedly expressed in the organ of Corti and spiral ligament after noise exposure. Neutralization of HMGB1 via anti-HMGB1 antibody treatment reduced the RNS-induced nitrative stress. These findings support our hypothesis that HMGB1 may have direct or indirect associations with noise-induced activation of iNOS.

NOX generates superoxide under stressful conditions and is recognized as one of the major sources of ROS in the noise-damaged cochlea [5]. Some members of the NOX family are upregulated by noise trauma, and NIHL can be alleviated by NOX inhibitors [25,35,36,37]. An interaction between the RAGE and TLR4 signaling pathways and NOX4 has been reported to generate ROS in several disease models [16,17,18,27]. Extracellular HMGB1, upon binding to the RAGE and TLR4 expressed in the inner ear, can further activate signaling cascades [33,38,39]. Our results presented here demonstrated an upregulated expression of cochlear NOX4 after noise exposure and a significant repression of this NOX4 activation by neutralization of HMGB1. Thus, the Nox4-derived ROS generation showed a strong association with HMGB1 expression; therefore, it might be regulated via some type of HMGB1-related signaling mechanism in the noise-exposed cochlea.

Repression of extracellular HMGB1 can reduce the inflammatory response in disease, thereby modulating HMGB1-associated immune dysfunction as well [40]. For this reason, HMGB1 has been recognized as a promising therapeutic target. Antibodies that neutralize HMGB1 confer protection against arthritis-related damage and tissue injury, colitis, ischemia, sepsis, endotoxemia, and systemic lupus erythematosus [10,12,13,14,40]. Treatment with anti-HMGB1 antibodies also diminishes ROS generation in the ischemia-reperfusion injury of brain, heart, liver, and kidney [41]. Recently, P5779, an HMGB1 inhibitor that targets the HMGB1-TLR4/MD-2 pathway to inhibit HMGB1-induced response, has been demonstrated to improve survival in experimental models of liver ischemia/reperfusion and sepsis [40]. Similarly, resveratrol, a wine polyphenol that activates surtuin-1 (SIRT1), was demonstrated to reduce inflammation by inhibiting HMGB1/TLR4 signaling in models of asthma and ischemic brain injury [42,43]. Glycyrrhizin, a naturally occurring anti-inflammatory and antiviral triterpene that directly binds to HMGB1, has been applied in the clinic to inhibit cytokine activities [44]. In the present study, our results explain in part the role of HMGB1 in noise-induced cochlear damage and hearing loss and may support the clinical application of HMGB1 inhibitors for the prevention of NIHL.

The blood–labyrinthine barrier (BLB), similar to the blood–brain barrier (BBB), can restrict the delivery of therapeutic agents from blood into the inner ear [45]. The mechanism of action of the HMGB1 inhibition via anti-HMGB1 Ab neutralization that underlies the current noise-exposed cochlea model remains to be clarified; however, a few speculative mechanisms have been proposed. One mechanism is based on an immunologic study on the inner ear by Mogi et al., who found that the immunoglobulins (IgG and albumin) in perilymph of the cochlea were mainly derived from infiltration from the blood vessels surrounding the perilymphatic space [46,47], suggesting that immunoglobulins can conquer the blood–labyrinth barrier. A similar finding was verified in the CNS by demonstrating that the large IgG molecule could cross the blood–brain barrier (BBB) of the guinea pig [48]. Therefore, delivery of anti-HMGB1 Abs via intravenous or intraperitoneal injections could achieve brain therapeutic benefits from hippocampal neuronal death and cognitive impairment in animal models [49].

A second possible mechanism involves entry of pre-treated anti-HMGB1 Abs in the blood vessels into the perilymph through altered permeability of the BLB following noise-induced trauma. Support for this mechanism in the BBB has been demonstrated by the observation that more anti-HMGB1 Abs enters the brain through the damaged BBB that results from a wide range of CNS diseases [50]. A third potential mechanism is through paracrine/autocrine regulatory mechanism via the release of HMGB1 from the spiral ligament, organ of Corti or spiral ganglion and its diffusion from the inner ear to the blood side, followed by neutralization by anti-HMGB1 Abs administered around the capillaries, thereby diminishing the level of cell-secreted HMGB1 that acts on nearby cells in a paracrine way [51]. Further experiments are needed in future to establish which models are most likely to explain the inhibition of HMGB1 in noise-exposed cochlea via anti-HMGB1 Ab neutralization.

## 5. Conclusions

We present the first evidence that HMGB1 signaling may participate in the cochlear oxidative stress induced by noise trauma. The upregulation of HMGB1 expression was associated with elevated generation of ROS/RNS following noise exposure. Manipulation of HMGB1 expression using anti-HMGB1 antibody pretreatment helped to reduce cochlear ROS/RNS generation, preserved more outer hair cells in the organ of Corti, and resulted in significantly less deterioration in the auditory threshold shifts associated with NIHL. This information suggests that targeting HMGB1 may represent a promising therapeutic approach for NIHL treatment in the future.

## Data Availability

All relevant data are included within the manuscript. The raw data are available on request from the corresponding author.
